# Enhanced Photocatalytic Paracetamol Degradation by NiCu-Modified TiO_2_ Nanotubes: Mechanistic Insights and Performance Evaluation

**DOI:** 10.3390/nano14191577

**Published:** 2024-09-29

**Authors:** Marco Pinna, Martina Zava, Tommaso Grande, Veronica Prina, Damiano Monticelli, Gianluca Roncoroni, Laura Rampazzi, Helga Hildebrand, Marco Altomare, Patrik Schmuki, Davide Spanu, Sandro Recchia

**Affiliations:** 1 Department of Science and High Technology, University of Insubria, Via Valleggio 11, 22100 Como, Italy; mzava@uninsubria.it (M.Z.); tgrande@studenti.uninsubria.it (T.G.); veronica.prina@uninsubria.it (V.P.); damiano.monticelli@uninsubria.it (D.M.); groncoroni1@uninsubria.it (G.R.); sandro.recchia@uninsubria.it (S.R.); 2 Dipartimento di Chimica, Università Degli Studi di Milano, Via Golgi 19, 20133 Milan, Italy; marco.pinna@unimi.it; 3 Department of Human Sciences and Innovation for the Territory, University of Insubria, via Sant’Abbondio 12, 22100 Como, Italy; laura.rampazzi@uninsubria.it; 4 Department of Materials Science WW4-LKO, Friedrich Alexander University of Erlangen Nuremberg, Martensstrasse 7, 91058 Erlangen, Germany; helga.hildebrand@fau.de (H.H.); patrik.schmuki@fau.de (P.S.); 5 Department of Chemical Engineering, MESA+ Institute for Nanotechnology, University of Twente, P.O. Box 217, 7500 AE Enschede, The Netherlands; m.altomare@utwente.nl; 6 Regional Center of Advanced Technologies and Materials, Šlechtitelů 27, 78371 Olomouc, Czech Republic

**Keywords:** photocatalysis, paracetamol, TiO_2_ nanotubes, water remediation, NiCu bimetallic catalysts, green chemistry, heterojunction, emerging pollutants

## Abstract

Anodic TiO_2_ nanotube arrays decorated with Ni, Cu, and NiCu alloy thin films were investigated for the first time for the photocatalytic degradation of paracetamol in water solution under UV irradiation. Metallic co-catalysts were deposited on TiO_2_ nanotubes using magnetron sputtering. The influence of the metal layer composition and thickness on the photocatalytic activity was systematically studied. Photocatalytic experiments showed that only Cu-rich co-catalysts provide enhanced paracetamol degradation rates, whereas Ni-modified photocatalysts exhibit no improvement compared with unmodified TiO_2_. The best-performing material was obtained by sputtering a 20 nm thick film of 1:1 atomic ratio NiCu alloy: this material exhibits a reaction rate more than doubled compared with pristine TiO_2_, enabling the complete degradation of 10 mg L^−1^ of paracetamol in 8 h. The superior performance of NiCu-modified systems over pure Cu-based ones is ascribed to a Ni and Cu synergistic effect. Kinetic tests using selective holes and radical scavengers unveiled, unlike prior findings in the literature, that paracetamol undergoes direct oxidation at the photocatalyst surface via valence band holes. Finally, Chemical Oxygen Demand (COD) tests and High-Resolution Mass Spectrometry (HR-MS) analysis were conducted to assess the degree of mineralization and identify intermediates. In contrast with the existing literature, we demonstrated that the mechanistic pathway involves direct oxidation by valence band holes.

## 1. Introduction

The improvements in the quality of life that humanity has experienced since the Industrial Revolution have been accompanied by a significant increase in global water pollution [1]. Over the past few decades, particular focus has been directed toward the study of the so-called emerging pollutants [2]. This category includes a wide range of molecules, such as pharmaceuticals [3], personal care products [4], pesticides [5], and plasticizers [6], which exert adverse effects on both human health and the environment [7]. These substances have been globally found in concentrations ranging from ng L^−1^ to μg L^−1^ in surface waters [8] due to anthropogenic sources such as sewage sludges, agricultural runoff, and industrial activities [6]. Nonetheless, most of these substances currently lack international regulations specifying maximum concentration levels in natural waters. The persistence of these molecules in treated waters underlines the inability of traditional wastewater treatments (e.g., sedimentation, flocculation, coagulation) to completely remove these pollutants [9]. Among them, paracetamol, the world’s most-used over-the-counter medication, has raised significant concern as an emerging pollutant [10,11]. In fact, due to the incomplete metabolization of the human body, large quantities of paracetamol enter the aquatic ecosystem, mostly through human excretions and hospital wastewater [12].

In this scenario, green and cost-effective removal strategies to be coupled with conventional techniques are highly looked-for. It should be underlined that such strategies are expected to work effectively for the abatement of low concentrations of paracetamol (below 10 mg L^−1^, normally observed in polluted waters), i.e., where conventional abatement methods fail.

Advanced Oxidation Processes (AOPs) based on heterogeneous photocatalysis, which allow the conversion of light energy to chemical energy, are promising for this purpose [13,14,15,16,17]. Photo-induced AOPs allow for the decontamination of waters by reaction of the target pollutant with in situ generated Reactive Oxygen Species (ROS) [18] or directly with photogenerated holes at the catalyst surface [19,20]. The main advantage of heterogeneous photocatalytic AOP over other AOP strategies (e.g., Electro-Fenton processes [21], ozonation [22], persulfate activated processes [23]) lies in milder process conditions and in the avoidance of additional chemicals and formation of sludges [20].

In the context of environmental photocatalysis, titanium dioxide (TiO_2_) continues to be the most studied material to date, thanks to its non-toxicity, stability against (photo)corrosion, low cost, and availability [24]. However, this material suffers from well-known drawbacks like high charge carrier recombination, which strongly limits its catalytic efficiency. One-dimensional nanostructures such as nanotubes have been widely employed to tackle this issue as they enable improved charge carrier separation and largely increased surface area [25]. To further improve the charge separation, the formation of heterojunctions with metals [26], metal oxides [27], and carbonaceous materials is widely employed [28]. Among these co-catalysts, noble metals have been successfully employed to promote the degradation of organic pollutants [29,30]. However, their high cost and low availability present critical constraints when considering the scalability of photocatalytic processes. For this reason, researchers have focused their attention on the use of earth-abundant non-noble metals (e.g., Ni, Cu, Fe), proving their relatively high efficiency for a fraction of the price of noble metals [31,32]. Recently, bimetallic co-catalysts, formed by combining these metals, have been under investigation as they enable the tuning of optical and electronic properties through composition control, leading to a significant enhancement of photocatalytic efficiency [33,34]. Among these materials, NiCu alloys have shown a remarkable synergistic effect toward photocatalytic water splitting and hydrogen evolution [35,36]. Such alloy co-catalysts enable a more efficient charge transfer compared with pure metal counterparts. This effect has been mostly related to an optimal work function shift and the presence of both Ni-rich and Cu-rich active sites synergistically responsible for different processes at the surface of the photocatalyst [37,38,39]. Despite the benefits associated with utilizing non-noble metals, only a few studies have explored pure Ni-modified [31] or Cu-modified [40] TiO_2_ catalysts (in powder form) for photocatalytic AOPs, while the absence of studies on photocatalysts modified with NiCu alloys for this purpose is noteworthy.

Thus, in this work, anodic TiO_2_ nanotubes decorated with layers of Ni, Cu or NiCu were employed in this study for the photocatalytic degradation of paracetamol in water. A systematic investigation was conducted to explore the influence of co-catalyst composition and loading. The aim was to identify the optimal photocatalyst and gain insights into the role of Ni and Cu in enhancing photocatalytic activity. Information on the reaction mechanism was gained through photocatalytic tests conducted in the presence of scavengers for hydroxyl radicals and holes, supplemented by High-Resolution Mass Spectrometry measurements.

## 2. Materials and Methods

### 2.1. TiO_2_ Nanotube Array Fabrication

Ti foils (99.7%, 0.127 mm thickness, Sigma Aldrich, St. Louis, MO, USA) were used as the starting materials for the fabrication of anodic TiO_2_ nanotube layers. Foils were cut to obtain slices with a 1.5 × 1 cm^2^ active area. After cutting, they were cleaned by sonication in acetone (Carlo Erba, Milano, Italy), isopropanol (Sigma Aldrich), ethanol (Sigma Aldrich), and ultrapure water in an ultrasonic bath (Sonorex, Bandelin, Berlin, Germany). Each sonication step was conducted for 15 min. Then, the cleaned Ti slices were dried under a nitrogen stream. Anodization was carried out in an ethylene glycol solution (99.5%, Carlo Erba) containing 0.09 M NH_4_F (96%, Thermo Scientific, Waltham, MA, USA) and 2% *v*/*v* ultrapure H_2_O. To perform the anodization, a two-electrode configuration was employed (Ti foil as anode and working electrode and a Ti mesh coated with a Pt layer as counter-electrode) with an applied potential of 40 V using a DC Power Supply (NPS1230W, Wanptek, Shenzhen, China). The as-formed TiO_2_ nanotube layers supported on Ti foils were rinsed and then soaked in ethanol overnight. Lastly, the amorphous nanotubes were annealed and crystalized in air at 450 °C for 1 h with a 5 °C min^−1^ ramp.

### 2.2. Sputter Deposition of Ni, Cu and NiCu Thin Films

To deposit thin films of metals onto the TiO_2_ substrate surface, a magnetron sputter-coater (Cressington 108auto, Cressington Scientific Instruments Ltd., Watford, UK) employing the following targets was used: 100Cu (99.60 at.% pure, Nanovision s.r.l., Brugherio, Italy), 100Ni (99.98 at.% pure, Nanovision s.r.l.), 25Ni75Cu (25 at.% Ni, 75 at.% Cu), 50Ni50Cu (50 at.% Ni, 50 at.% Cu), and 75Ni25Cu (75 at.% Ni, 25 at.% Cu). The NiCu alloy sputter targets were provided by Hauner Metallische Werkstoffe (Röttenbach, Germany). Samples are herein labeled by the nominal thickness, and the chemical composition of the co-catalyst layer, e.g., (20 nm) 50Ni50Cu-TiO_2_ is a sample decorated with a 20 nm-thick layer of the 50Ni50Cu alloy. All details about tested photocatalytic materials are summarized in Table S1. Due to the different sputter yields of Ni, Cu, and their alloys, different currents were applied to achieve comparable thin film deposition times. Specifically, the current used during sputtering was set at 20 mA for Cu, 30 mA for all the NiCu alloys, and 40 mA for pure Ni. Independently of the target being used, all depositions were carried out under Ar with a chamber pressure always set at 10^−1^ mbar. To calibrate the sputter deposition process, the nominal thickness of the metal films obtained at fixed times and currents was determined using sputter coating square-shaped glass substrates (1.8 × 1.8 cm) and then selectively dissolving the Ni, Cu, or NiCu metal film with 2 mL of ultrapure nitric acid (obtained by a sub-boiling distillation system (Duopur, Milestone [41]), under sonication for 30 min at 30 °C, followed by 1 h of agitation using an orbital shaker (SSL1, Stuart). After proper dilution, analysis by Inductively Coupled Plasma Mass Spectrometry (ICP-MS) (iCAP-Q, ThermoScientific) of the obtained solutions was conducted to determine the amount of sputtered metal. A summary of the employed sputtering conditions and calibration is reported in Table S2. The overall photocatalyst’s fabrication, including the growth of TiO_2_ nanotube arrays and sputter deposition of Ni, Cu, and NiCu thin films, is schematically depicted in Figure S1.

### 2.3. Characterization of Materials

The crystallographic features of the different materials were investigated using X-ray Diffraction (XRD) with a Siemens D500 (Siemens AG, Munich, Germany) employing Cu Kα radiation at 40 kV and 40 mV and equipped with 1 mm slits. The measurements were conducted employing Bragg-Brentano geometry, a step size of 0.03° (2θ), and a scanning speed of 0.06° s^−1^. The samples were attached to an aluminum sample holder using carbon tape.

The morphology of both pristine and NiCu-decorated TiO_2_ nanotubes was examined with Scanning Electron Microscopy (SEM) using a Philips XL30 (Philips A.V.) at 30 kV under high vacuum conditions. The samples were attached to an SEM stub using conductive carbon tape.

Information regarding the oxidation state of Ni and Cu for fresh photocatalysts was obtained through X-ray Photoelectron Spectroscopy (XPS) using a PHI instrument from Physical Electronics Inc. Peak positions were calibrated with respect to the C1s peak at 284.8 eV. Fitting was carried out using CasaXPS 2.2.25 (Casa Software Ltd., Teignmouth, UK).

Diffuse reflectance UV-visible spectroscopy (DR-UV) measurements on solid samples were performed using a Shimadzu UV-2600i spectrometer.

### 2.4. Photocatalytic Tests and Mechanistic Studies

The photocatalytic experiments were carried out by immersing the photocatalyst layers in a stirred solution containing 10 mg L^−1^ paracetamol (98%, Thermo Scientific) in ultrapure water. To ensure transparency to UV radiation, a quartz test tube (volume capacity ~15 mL) was employed. In the experimental setup, 12 mL of solution was exposed to irradiation. To achieve equilibration, the system was kept in dark condition for 30 min prior to illumination. Then, UV irradiation was provided using a UV LED source (λ = 365 nm, UVWave) with a power density of 85 mW cm^−2^. To simulate solar radiation, a Sun 2000 Solar Simulator (Abet Technologies, Milford, CT, USA, calibrated at 85 mW cm^−2^) equipped with an AM 1.5 G filter was used as the vertical light source: to carry out the experiments in a side-illumination configuration, a totally reflecting Al mirror was used to deflect the light beam. To quantitatively assess the photocatalytic efficiency, the solution was analyzed by sampling 200 μL every 30 min. Paracetamol determination was performed using a High-Performance Liquid Chromatography system equipped with a Diode Array Detector (HPLC-DAD) (SPD-M40, Shimadzu, Kyoto, Japan) in isocratic mode with a 1:1 acetonitrile-acidified water (0.1% *v*/*v* Trifluoroacetic Acid) mobile phase (flow rate = 0.8 mL min^−1^). A C18 column (column size 150 × 4 mm, particle size 25 µm, column temperature 40 °C, Interchrom, Montluçon, France) was employed for separation. To avoid interferences from the eluent itself, quantifications were carried out at 269 nm. All photocatalytic experiments were conducted in triplicate, and error bars represent one standard deviation.

The degree of mineralization was assessed by determining the Chemical Oxygen Demand (COD) with a spectroscopic kit from Merck and a Nova 60a spectrometer. This analytical method offers a quantification of the level of mineralization achieved, through the determination of the equivalent amount of oxygen (expressed as mg L^−1^ of O_2_) necessary to achieve the complete mineralization of organic compounds in water solution.

The degree of mineralization was calculated according to Equation (1):

Mineralization (%) = [(COD_0_ − COD_T_)/COD_0_] × 100, (1)

in which COD_0_ is the value obtained for a fresh 10 mg L^−1^ solution (i.e., not exposed to UV radiation in the presence of a photocatalyst), and COD_T_ is obtained after different durations of the photocatalytic treatment.

Reaction intermediates were qualitatively identified by analyzing paracetamol solutions at specific time intervals during the photocatalytic treatment using High-Resolution Mass Spectrometry in Direct Infusion ElectroSpray Ionization (ESI) mode (Orbitrap Exploris 120, Thermo Scientific) in the 40–300 m z^−1^ range, employing both positive and negative ionization modes. Aliquots of the reaction solutions were sampled employing a 10 µL min^−1^ flow and ionized, applying respective potentials of +3.3 kV and −3.2 kV. Brute formulas were automatically detected using the software Freestyle (ThermoScientific), which employs a proprietary algorithm based on the main peak and isotope ratios.

Mechanistic studies were complemented by photocatalytic tests in the presence of scavengers. Specifically, experiments were carried out by employing a 10 mg L^−1^ paracetamol solution containing 1 g L^−1^ of either tert-Butyl alcohol (99%, Merck, Darmstadt, Germany) or formic acid (99%, Carlo Erba) to selectively react with hydroxyl radicals or surface holes, respectively [42,43].

## 3. Results and Discussion

### 3.1. Photocatalysts Characterization

**Figure 1:**
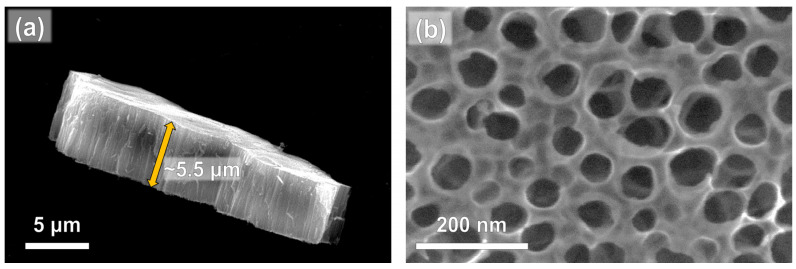
(**a**) SEM cross-sectional and (**b**) top-view images of pristine TiO_2_ nanotube arrays.

Anodic high aspect-ratio TiO_2_ nanotubes, shown in Figure 1, were utilized in this work. The obtained nanotubes exhibit an average length of 5.5 μm (Figure 1a) and an average inner diameter of 80 nm (Figure 1b). The anodization conditions were selected to specifically fabricate nanotubes with an optimized morphology in terms of efficient light absorption and charge carrier separation [44].

Following anodization, the amorphous nanotubes underwent annealing in air at 450 °C for 1 h to induce TiO_2_ crystallization. X-ray Diffraction (XRD) analysis confirms the occurrence of this process (Figure S2). Peaks observed at 25.4°, 40.4°, 48.1°, 54.0°, and 55.1° were assigned, respectively, to the 101, 004, 200, 105, and 211 anatase crystal planes [45]. Peaks at 37.9°, 38.4°, 40.3°, and 50.3° were attributed to the 100, 002, 101, and 102 hexagonal titanium crystal planes (Ti metallic support) [46]. Finally, the peak at 44.6 ° was attributed to the 200 crystal plane reflection for metallic Al (sample holder) [47].

**Figure 2:**
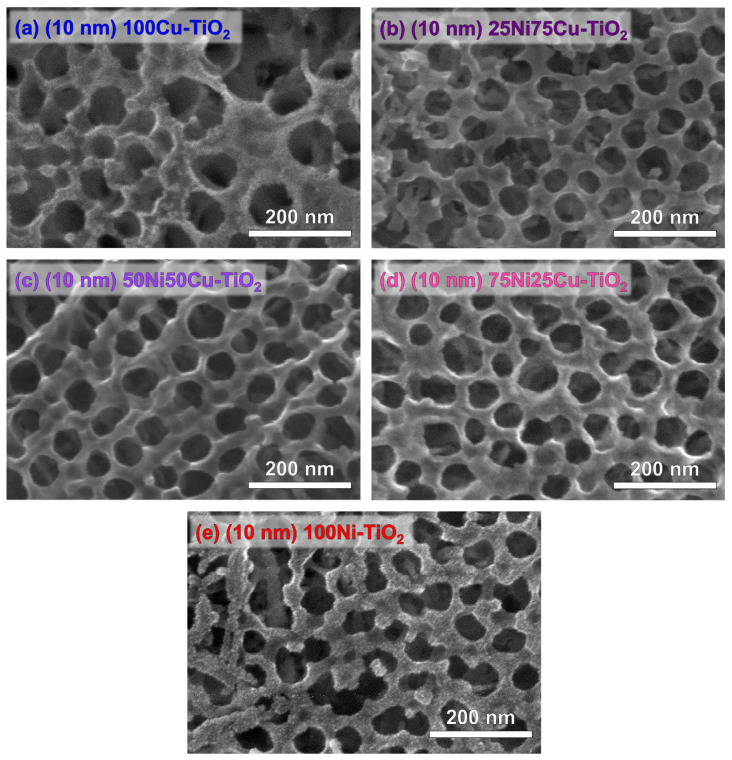
Top-view SEM micrographs of (**a**) 100Cu-TiO_2_, (**b**) 25Ni75Cu-TiO_2_, (**c**) 50Ni50Cu-TiO_2_, (**d**) 25Ni75Cu-TiO_2_, and (**e**) 100Ni-TiO_2_. All samples were obtained by sputtering a nominal thickness of 10 nm.

Figure 2 shows top-view SEM micrographs of annealed TiO_2_ nanotubes sputtered with 10 nm of 100Cu, 100Ni, and NiCu alloys (three different compositions: 25Ni75Cu, 50Ni50Cu, and 75Ni25Cu). Pure metal Ni and Cu thin films (Figure 2a,e) are composed of small nanoparticles (size < 10 nm), while the alloyed co-catalyst (Figure 2b–d) presents a more conformal film morphology, apparently without significant differences for different NiCu composition.

Similarly, morphological changes induced by different loadings of the 50Ni50Cu alloy loadings were analyzed by SEM. As shown in Figure S3, increasing the amount of alloyed co-catalyst on the surface of the semiconductor from a nominal thickness of 1 nm (Figure S3a) to 10 nm (Figure S3b) does not lead to any clearly visible difference. However, when depositing a thicker film (20 nm, Figure S3c), the surface is covered with a layer of small nanoparticles. Finally, when sputtering a nominal thickness of 25 nm (Figure S3d), the sputtered nanoparticles tend to agglomerate into larger particles, especially in the proximity of the triple points of the ordered nanotubular arrays.

Samples decorated with an increasing amount of sputtered 50Ni50Cu alloy were also subjected to XRD analysis (Figure S2). Specifically, nominal thicknesses of 5, 10, and 20 nm were selected. As expected, due to the very low amount of sputtered material (most likely below the instrumental limit of detection), no signals for Ni, Cu, or their alloy were detected: the absence of such signals could also be related to the amorphous nature of the sputtered thin film [36,48].

**Figure 3:**
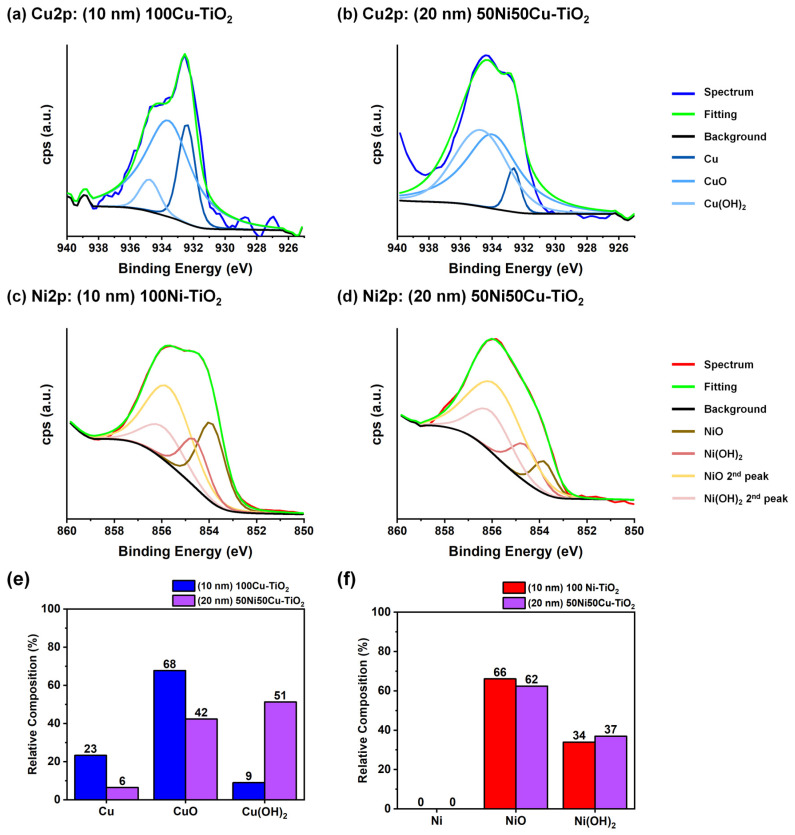
(**a**) HR-XPS fitting at the Cu2p edge for (10 nm) 100Cu-TiO_2_ and (**b**) (20 nm) 50Ni50Cu-TiO_2_. In (**c**), HR-XPS fitting at the Ni2p edge for (10 nm) 100Ni-TiO_2_ and (**d**) (20 nm) 50Ni50Cu-TiO_2_. In (**e**), copper relative composition of the 100Cu-TiO_2_ (blue) and (20 nm) 50Ni50Cu-TiO_2_ (purple), while in (**f**), nickel relative composition of the 100Cu-TiO_2_ (red) and (20 nm) 50Ni50Cu-TiO_2_ (purple).

The oxidation state of Cu and Ni in pure Cu, pure Ni and 50Ni50Cu co-catalysts was examined using High-Resolution XPS analysis in the Cu2p and Ni2p regions. Fittings for all recorded spectra are reported in Figure 3. All Cu-containing materials (Figure 3a,b) exhibit a variable composition in terms of Cu speciation as testified by the presence of peaks corresponding to Cu (932.6 eV), CuO (933.8 eV), and Cu(OH)_2_ (934.7 eV) [49]. In both pure metals- and alloy-decorated materials, copper is predominantly present in the Cu(II) oxidation state. The main distinction is a higher fraction of oxidized Cu in the alloyed material (Figure 3e). This discrepancy can be reasonably attributed to the different sputtering yields and rates of the two materials. Specifically, due to the magnetic nature and the higher susceptibility to oxidation of Ni, a longer sputtering time is necessary for the NiCu-decorated material preparation compared with Cu-decorated ones [50]. Consequently, due to the relatively high background pressure (10^−1^ mbar) in the sputtering chamber, more oxygen can be incorporated into the sputtered thin film during the deposition process [51,52,53]. In contrast, no difference is observable for Ni speciation (Figure 3c,d): in both materials, Ni appears predominantly as NiO (855.7 eV) [54] due to its tendency to oxidize compared with Cu when exposed to air [55].

The oxidized nature of the thin NiCu film was confirmed by the HR-XPS analysis in the O1s region. The two main components of the spectrum appear to be lattice oxygen atoms, identified by the peak at 529.7 eV and surface hydroxyl groups (531.3 eV). A third component, a wider and smaller peak at 533 eV, was attributed to the presence of adsorbed H_2_O on the surface of the sample, reasonably due to exposition to ambient conditions. The observed peaks are coherent with the reported literature on metal oxides [56].

Based on the XPS findings, we opted to explore the potential sensitization to visible-range light facilitated by surface Cu or NiCu oxides. This phenomenon is well-documented for CuO and is attributed to its narrow band gap (1.3–1.7 eV) [57], whereas no clear evidence is provided for mixed oxides. To assess the light absorption properties of different samples, DR-UV measurements were conducted. As observed in Figure S5, no significant difference is evident between pristine and decorated TiO_2_ in the 200–800 nm range. Tauc’s plots (obtained by processing the data using the Kubelka–Munk function) were used to estimate the band gap energies (Figure S5 inset) [58,59,60]: both pristine and decorated materials exhibit an E_g_ of approximately 3.2 eV, a value commonly reported for anatase [25], highlighting the absence of sensitization toward the visible range. For this reason, we decided to employ UV irradiation at 365 nm for all photocatalytic experiments.

### 3.2. Photocatalytic Experiments: The Effect of the Co-Catalyst

Before conducting photocatalytic tests, the photostability of paracetamol under illumination conditions was evaluated (i.e., λ = 365 nm, irradiance = 85 mW cm^−2^). A 10 mg L^−1^ paracetamol solution was irradiated for 3 h in the absence of photocatalysts. Only negligible photodegradation of paracetamol was observed (<5%). The adsorption of paracetamol onto pristine TiO_2_, as well as onto decorated materials, was ruled out since, after three hours of experiments under dark conditions, no decrease in paracetamol concentrations was observed. Therefore, it can be concluded that any decrease in paracetamol concentration is attributable to the activity of the photocatalyst under UV light.

**Figure 4:**
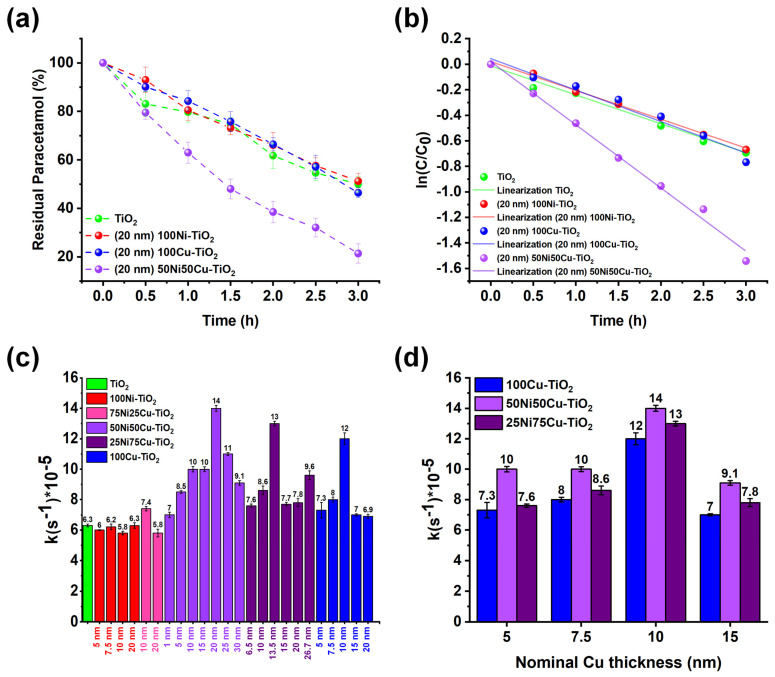
In (**a**), the kinetic tests for paracetamol photocatalytic degradation for TiO_2_ (green), (20 nm) 100Ni-TiO_2_ (red), (20 nm) 100Cu-TiO_2_ (blue), and (20 nm) 50Ni50Cu-TiO_2_ (purple); in (**b**), first-order kinetic linearization of the reported kinetic tests. In (**c**), kinetic constants obtained by first-order linearization for the differently tested photocatalysts, while in (**d**), kinetic constants obtained for Cu-rich (Cu atomic fraction > 50%) categorized by nominal loading of Cu.

Benchmarking of the photocatalytic degradation was therefore performed. The investigation focused on the co-catalyst thin-film composition and thickness. In accordance with the literature [61,62], a first-order kinetic linearization (Figure 4a,b) was applied, and the resulting kinetic constants are reported in Figure 4c for all the tested materials.

An initial observation regarding the impact of the sputtered thin film composition is evident: Cu-rich materials (Cu content ≥ 50%) exhibit significantly higher performance than Ni-rich ones (showing no apparent improvements compared with pristine TiO_2_), underscoring Cu as the primary contributor in enhancing the photocatalytic efficiency. A closer examination reveals an optimal thickness for each type of co-catalyst: a bell-shaped profile in terms of photocatalytic performance is observed depending on the thickness of the sputtered co-catalyst film. This trend is often observed and can be interpreted considering that the deposition of larger amounts of co-catalyst leads to a decrease in photocatalytic efficiency, likely due to a reduction in available TiO_2_ surface, while small amounts of co-catalyst do not maximize the charge transfer.

Interestingly, the three best-performing materials ((20 nm) 50Ni50Cu-TiO_2_, (13.5 nm) 25Ni75Cu-TiO_2_, and (10 nm) 100Cu-TiO_2_) are characterized by the same Cu loading (nominal thickness of Cu = 10 nm). Nevertheless, NiCu alloys show superior photocatalytic performances over pure Cu, with (20 nm) 50Ni50Cu-TiO_2_ being the best-performing material (~80% paracetamol abatement over a 3 h irradiation step, see Figure 4a), showing a 2.2-times higher kinetic constant in comparison to pristine TiO_2_. This evidence suggests a synergistic effect of Ni and Cu in enhancing the photocatalytic activity, likely attributed to improved charge carrier dynamics in mixed Ni-Cu systems. Specifically, as reported in the literature, the decoration of TiO_2_ with Ni and Cu can lead to the formation of heterojunctions: in these systems, photogenerated charge carriers are spatially separated, leading to improved charge transfer to reactants and an overall lower recombination rate [63,64,65,66]. Moreover, enhancement of transfer to reactants has been observed when employing mixed Cu and Ni phases in comparison to pure metals [67,68]. This feature is clear when plotting the effect of the nominal Cu loading (expressed in terms of thickness) on the photocatalytic activity of Cu-, 25Ni75Cu-, and 50Ni50Cu-TiO_2_ (Figure 4d): the efficiency toward paracetamol degradation at the same Cu content is always higher when Ni is present, confirming the synergistic effect in the bimetallic system.

**Figure 5:**
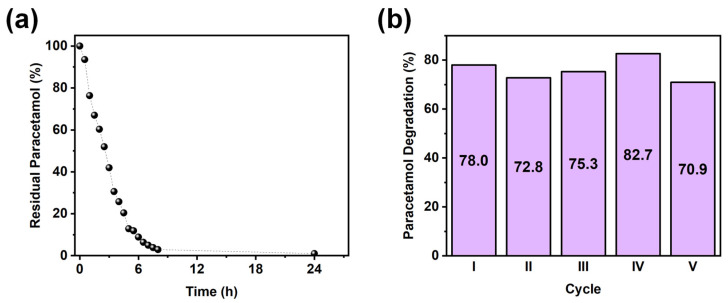
(**a**) Kinetic profile obtained using (20 nm) 50Ni50Cu-TiO_2_ photocatalyst in a 24 h-long photocatalytic experiment. (**b**) Photocatalytic efficiency for up to five consecutive catalytic cycles obtained with the same material.

After identifying the best-performing material, additional tests were conducted to evaluate (i) the required time for complete paracetamol degradation and (ii) the reusability of the photocatalyst. Complete paracetamol degradation was investigated by carrying out a long irradiation test lasting up to 24 h. As depicted in Figure 5a, complete paracetamol degradation was achieved in approximately 8 h. Regarding reusability, five consecutive abatement tests were conducted using the same material without any specific regeneration treatment between each experiment: the photocatalyst was simply rinsed with ultrapure water, dried under a nitrogen stream, and then reused. As shown in Figure 5b, no significant efficiency loss can occur for up to five cycles, highlighting the stability of the material.

Finally, to confirm the absence of sensitization toward visible light, photocatalytic degradation tests under simulated solar radiation were performed using pristine TiO_2_ and (20 nm) 50Ni50Cu-TiO_2_ (Figure S6). In both cases, the kinetic constants are lower compared with those obtained under UV irradiation due to the lower UV photon flux absorbed. More specifically, a constant of 3.77∙10^−5^ s^−1^ was observed for pristine TiO_2_, while a constant of 7.87∙10^−5^ s^−1^ was obtained for (20 nm) 50Ni50Cu-TiO_2._ The overall enhancement for the NiCu-modified photocatalyst is approximately two times compared with pristine TiO_2_, i.e., close to the improvement calculated when employing UV radiation. This further confirms that the use of a 50Ni50Cu alloy does not induce sensitization to visible light: varying the incident light spectrum does not lead to any additional enhancement when comparing NiCu-modified TiO_2_ with pristine TiO_2_ photocatalysts.

### 3.3. Mechanistic Insights

To gain insights into the photocatalytic degradation mechanism of paracetamol, it is necessary to determine the active species responsible for the reaction.

For TiO_2_ based photocatalysts, hydroxyl radicals (OH∙), generated by reaction of water molecules with VB holes according to Equation (2), are generally reported in the literature as the main responsible for paracetamol oxidation [10,62].

h^+^_VB_ + H_2_O_(l)_ →H^+^_(aq.)_ + OH^∙^_(aq.)_ (2)

However, the direct oxidation of paracetamol at the photocatalyst surface by VB holes is also reported in the literature [69].

**Figure 6:**
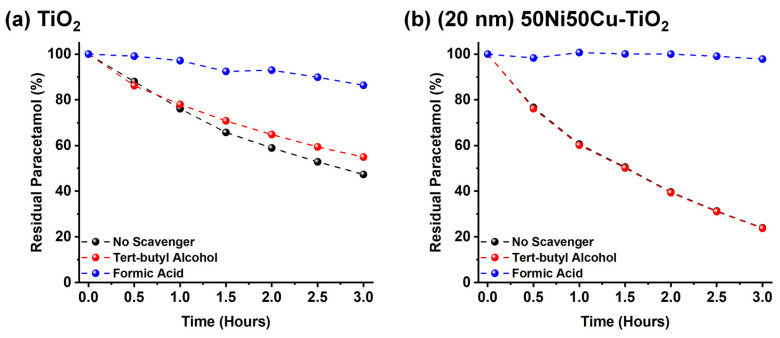
Photocatalytic test conducted without any scavenger (black) and in the presence of tert-butyl alcohol (red) or formic acid (blue) for (**a**) TiO_2_ and (**b**) (20 nm) 50Ni50Cu-TiO_2_.

As NiCu-modified TiO_2_ is applied for the first time for this application, kinetic tests were conducted to unambiguously determine whether VB holes or hydroxyl radicals primarily drive the photocatalytic process. We carried out photocatalytic experiments in the presence of scavengers, i.e., tert-butyl alcohol or formic acid, which are known to selectively react with hydroxyl radicals or valence band holes, respectively [42,43]. As reported in Figure 6, no significant loss in photocatalytic activity can be observed when employing 1 g L^−1^ of tert-butyl alcohol for both pristine (Figure 6a) and (20 nm) 50Ni50Cu decorated TiO_2_ (Figure 6b) suggesting that, contrarily to the reported literature, OH∙ radicals do not play a significative role in the photocatalytic degradation of paracetamol with TiO_2_ or NiCu-TiO_2_. On the contrary, when the reaction is carried out in the presence of 1 g L^−1^ of formic acid (hole scavenger), the degradation rate is significantly reduced and, especially in the case of NiCu-TiO_2_, no paracetamol degradation is observed after 3 h. Thus, based on this evidence, we can conclude that the photocatalytic degradation of paracetamol is mediated by surface valence band holes, which stands in contrast to the mechanisms generally reported and hypothesized in the literature for TiO_2_-based materials, where radical species, such as hydroxyl radicals, are typically considered the primary drivers of degradation [70,71].

By taking into consideration the oxidized nature of the thin metal film (discussed in detail in Section 3.2), as well as the evidence achieved by scavenger experiments, we propose that direct oxidation by surface valence band holes is the mechanism leading to paracetamol photocatalytic degradation. Specifically, we propose that photogenerated VB holes migrate from TiO_2_ toward the co-catalyst, thanks to the band alignment between TiO_2_ and Ni and Cu oxides [64,65]. Of course, as pristine TiO_2_ also possesses a remarkable activity toward paracetamol degradation, it is reasonable to suggest that paracetamol partially reacts directly with TiO_2_, even in NiCu-TiO_2_ systems. However, considering the large surface coverage of TiO_2_ active sites caused by the presence of the NiCu layer (see Figure 2), the direct decomposition on TiO_2_ sites is expected to be minimal when NiCu-TiO_2_ systems are employed. The reaction between holes and water, leading to hydroxyl radicals, cannot be excluded based on our data, but as previously reported, these reactive species do not seem to play a role in paracetamol degradation.

Finally, we investigated the degradation products. We specifically studied whether the degradation of paracetamol resulted in complete mineralization (i.e., oxidation to CO_2_) or the formation of intermediates (not detected by HPLC-DAD). Preliminary Chemical Oxygen Demand (COD) tests were conducted on a fresh paracetamol solution, as well as after 3 and 24 h of UV irradiation in the presence of the best performing material, i.e., (20 nm) 50Ni50Cu-TiO_2_.

As reported in Table 1, the observed degree of mineralization is consistently lower than the percentage of paracetamol degraded.

After 24 h of illumination (resulting in complete degradation of paracetamol), only 80% of full mineralization is achieved. Therefore, it can be concluded that some intermediates that are formed during the photocatalytic degradation of paracetamol are not completely oxidized to CO_2_ (at least within 24 h of irradiation).

To obtain information about the nature of such intermediates, High-Resolution Mass Spectrometry analysis was conducted. Samples were collected after 3, 8, and 24 h of UV illumination and analyzed in both positive and negative ElectroSpray Ionization (ESI+ and ESI). The detected species are summarized in Table S2 in the Supporting Information.

**Figure 7:**
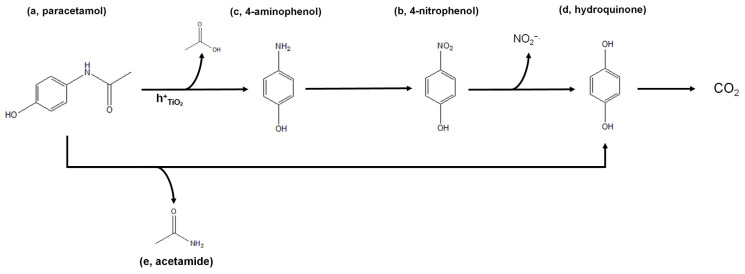
Schematic representation of the photocatalytic degradation of paracetamol.

Considering the species identified using HR-MS and the data reported in the literature [19,69], we proposed the photocatalytic degradation mechanism depicted in Figure 7. Oxidation by valence band holes on the surface of the photocatalyst can lead to the diacylation of paracetamol (intermediate a), resulting in the formation of 4-aminophenol (intermediate b). Subsequently, due to the high oxidant environment, intermediate b is oxidized to 1–4 nitrophenol (intermediate c). The loss of NO_2_^−∙^ radical then leads to the formation of hydroquinone (intermediate d). Another possible route is obtained by the loss of acetamide, leading to the direct formation of hydroquinone. Due to the unselective nature of radicals, both these processes can occur simultaneously, as evidenced by the presence of both 4-aminophenol and acetamide (intermediate e). Eventually, hydroquinone is oxidized to CO_2_. In any case, we cannot exclude the presence of undetected intermediate products between these two species. These molecules might not be detectable due to either presenting a nominal mass lower than 60 m z^−1^ (i.e., the cutoff value of the employed analytical method) or having a concentration below the instrumental LOD.

## 4. Conclusions

This study addressed the pressing issue of water pollution caused by pharmaceutical products, particularly focusing on paracetamol, a widely used medication with adverse environmental effects. Utilizing heterogeneous photocatalysis, our study aimed to investigate the effectiveness of a noble metal-free TiO_2_-based photocatalyst for the degradation of paracetamol. Anodic TiO_2_ nanotubes modified with conformal layers of Ni, Cu, or NiCu alloys were systematically investigated for the first time in view of their photocatalytic activity toward paracetamol oxidation. We found that Cu-rich materials exhibited superior performance over pristine and Ni-decorated TiO_2_ photocatalysts, with optimal thicknesses of the co-catalyst layer being critical for maximizing photocatalytic efficiency. Notably, the synergy between Ni and Cu in bimetallic NiCu layers significantly improved the photocatalytic activity with respect to pure Cu and Ni counterparts: this enhancement is attributed to improved charge transfer to reactants compared with the use of pure metals thanks to the formation of a heterojunction. We found that 20 nm-thick NiCu layers sputtered on ∼5.5 μm-thick TiO_2_ nanotube layers provide the highest efficiency for paracetamol degradation, showing a kinetic constant 2.2 times higher than that of pristine TiO_2_ nanotubes and leading to the complete degradation of 10 mg/L of paracetamol in around 8 h under UV light (12 mL of water volume treated). We also clearly demonstrated that direct oxidation by valence band holes is the main driving force behind paracetamol photocatalytic degradation, contrasting with the existing literature, where the degradation is typically attributed to radical species, such as hydroxyl radicals. Finally, the presence of residual intermediates was confirmed by High-Resolution Mass Spectrometry.

Future work will focus on applying the newly developed photocatalysts to other AOPs, such as the simultaneous degradation of active pharmaceuticals ingredients (APIs), as well as their optimization for use under real-life conditions.

## Figures and Tables

**Table 1:** COD analysis results from paracetamol at different irradiation times in the presence of 20 nm 50Ni50Cu-TiO_2_, with relative mineralization degree.

Irradiation Time (hours)	COD (O_2_ mg L^−1^)	Degraded Paracetamol	Mineralization Degree
0	22.4 mg L^−1^	0%	0%
3	14.4 mg L^−1^	78%	36%
24	4.7 mg L^−1^	98.8%	80%

## Data Availability

The raw data supporting the conclusions of this article will be made available by the authors on request.

## Supplementary Materials

The following supporting information can be downloaded at https://www.mdpi.com/article/10.3390/nano14191577/s1, Figure S1: Scheme of the photocatalyst’s fabrication; Figure S2: XRD patterns; Figure S3: SEM top-view images of NiCu-TiO_2_ with NiCu layers of varying thickness; Figure S4: HR-XPS spectrum and fitting in the O1s region for the (20 nm) 50Ni50Cu-TiO_2_ sample; Figure S5: DR-UV-vis absorption spectra; Figure S6: Kinetics test performed under simulated solar radiation; Table S1: Photocatalytic materials information; Table S2: Calibration information for sputtering deposition; Table S3: HR-MS results at different UV irradiation times.
