# Supplementary material for: Enhanced Photocatalytic Paracetamol Degradation by NiCu-Modified TiO2 Nanotubes: Mechanistic Insights and Performance Evaluation
Source: Nanomaterials (Basel). 2024 Sep 29;14(19):1577. doi: 10.3390/nano14191577 (PMC11477857; doi:10.3390/nano14191577)
Supplement: Supplementary file 1 [file nanomaterials-14-01577-s001.zip › nanomaterials-3225572-supplementary.pdf]

Supplementary Materials

Article

Enhanced Photocatalytic Paracetamol Degradation by NiCu-Modified TiO_2 Nanotubes: Mechanistic Insights and Performance Evaluation

Marco Pinna [1,2], Martina Zava [1], Tommaso Grande [1], Veronica Prina [1], Damiano Monticelli [1], Gianluca Roncoroni [1], Laura Rampazzi [3], Helga Hildebrand [4], Marco Altomare [5], Patrik Schmuki [4,6], Davide Spanu [1,*], Sandro Recchia [1]

1 Department of Science and High Technology, University of Insubria, Via Valleggio 11, 22100 Como, Italy; mzava@uninsubria.it (M.Z.); tgrande@studenti.uninsubria.it (T.G.); veronica.prina@uninsubria.it (V.P.); damiano.monticelli@uninsubria.it (D.M.); groncoroni1@uninsubria.it (G.R.); sandro.recchia@uninsubria.it (S.R.)

2 Dipartimento di Chimica, Università degli Studi di Milano, Via Golgi 19, Milan 20133, Italy; marco.pinna@unimi.it

3 Department of Human sciences and Innovation for the Territory, University of Insubria, via Sant'Abbondio 12, Como 22100, Italy; laura.rampazzi@uninsubria.it

4 Department of Materials Science WW4-LKO, Friedrich Alexander University of Erlangen Nuremberg, Martensstrasse 7, 91058 Erlangen, Germany; helga.hildebrand@fau.de (H.H); patrik.schmuki@fau.de (P.S.)

5 Department of Chemical Engineering, MESA+ Institute for Nanotechnology, University of Twente, P.O. Box 217, 7500 AE Enschede, The Netherlands; m.altomare@utwente.nl

6 Regional Center of Advanced Technologies and Materials, Šlechtitelů 27, Olomouc, 78371 Czech Republic

* Correspondence: davide.spanu@uninsubria.it; Tel.: +39 0312386428

Table S1. Detailed information regarding all photocatalytic materials used in this work.

**Sample**	**Thin-film thickness (nm)**	**Ni content (%)**	**Cu content (%)**
TiO_2	0	0	0
(5 nm) 100Ni-TiO_2	5	100	0
(7.5 nm) 100Ni-TiO_2	7.5	100	0
(10 nm) 100Ni-TiO_2	10	100	0
(20 nm) 100Ni-TiO_2	20	100	0
(10 nm) 75Ni25Cu-TiO_2	10	75	25
(20 nm) 75Ni25Cu-TiO_2	20	75	25
(1 nm) 50Ni50Cu-TiO_2	1	50	50
(5 nm) 50Ni50Cu-TiO_2	5	50	50
(10 nm) 50Ni50Cu-TiO_2	10	50	50
(15 nm) 50Ni50Cu-TiO_2	15	50	50
(20 nm) 50Ni50Cu-TiO_2	20	50	50
(25 nm) 50Ni50Cu-TiO_2	25	50	50
(30 nm) 50Ni50Cu-TiO_2	30	50	50
(6.5 nm) 25Ni75Cu-TiO_2	6.5	25	75
(10 nm) 25Ni75Cu-TiO_2	10	25	75
(13.5 nm) 25Ni75Cu-TiO_2	13.5	25	75
(15 nm) 25Ni75Cu-TiO_2	15	25	75
(20 nm) 25Ni75Cu-TiO_2	20	25	75
(26.7 nm) 25Ni75Cu-TiO_2	26.7	25	75
(5 nm) 100Cu-TiO_2	5	0	100
(7.5 nm) 100Cu-TiO_2	7.5	0	100
(10 nm) 100Cu-TiO_2	10	0	100
(15 nm) 100Cu-TiO_2	15	0	100
(20 nm) 100Cu-TiO_2	20	0	100

Table S2. Experimental conditions and obtained calibration curves for the different sputter targets employed.

**Metal target**	**Current**	**Chamber pressure**	**Calibration Curve (y: nominal thickness in nm x: time in seconds)**
Cu	20 mA	10^{-1} mbar	$y = 0.0304x – 0.5239$ (R^2=0.9905)
Ni	40 mA	10^{-1} mbar	$y = 0.0147x – 0.0664$ (R^2=0.9991)
25Ni75Cu	30 mA	10^{-1} mbar	$y = 0.1558x – 7.0278$ (R^2=0.9974)
50Ni50Cu	30 mA	10^{-1} mbar	$y = 0.0274x – 0.2718$ (R^2=0.9887)
75Ni25Cu	30 mA	10^{-1} mbar	$y = 0.0101x + 0.0266$ (R^2=0.9981)

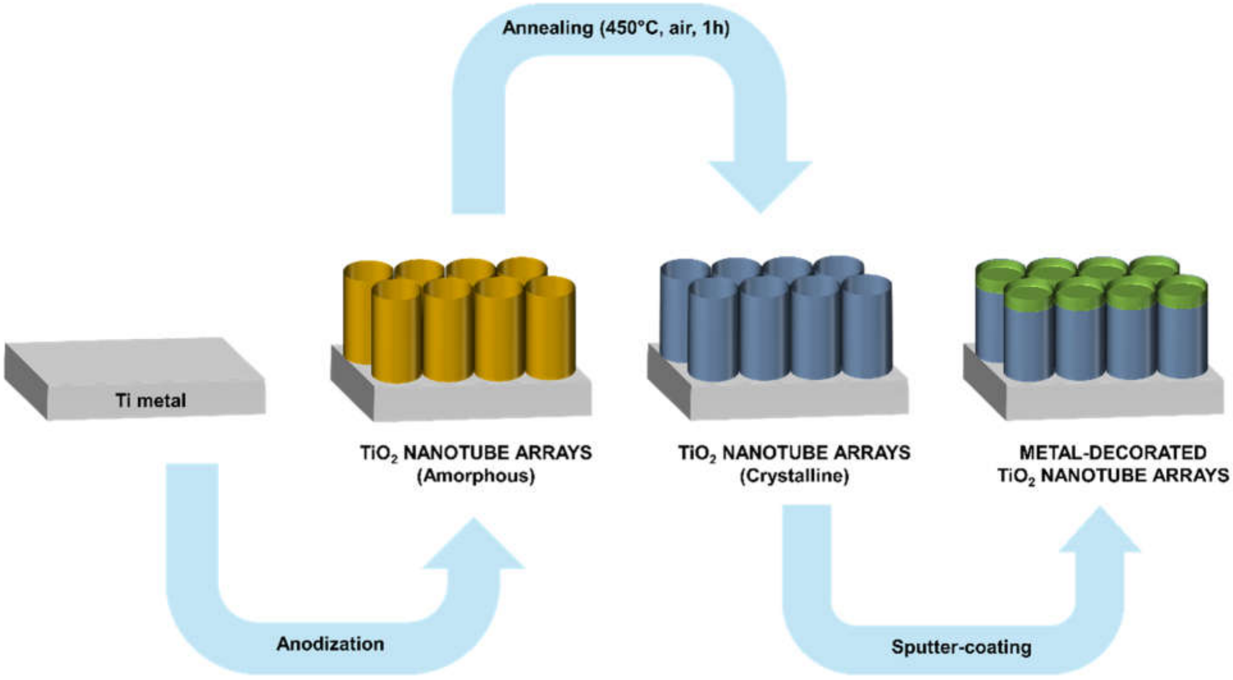

Figure S1. Scheme of the overall photocatalyst's fabrication including the growth of TiO_2 nanotube arrays and sputter deposition of Ni, Cu and NiCu thin films.

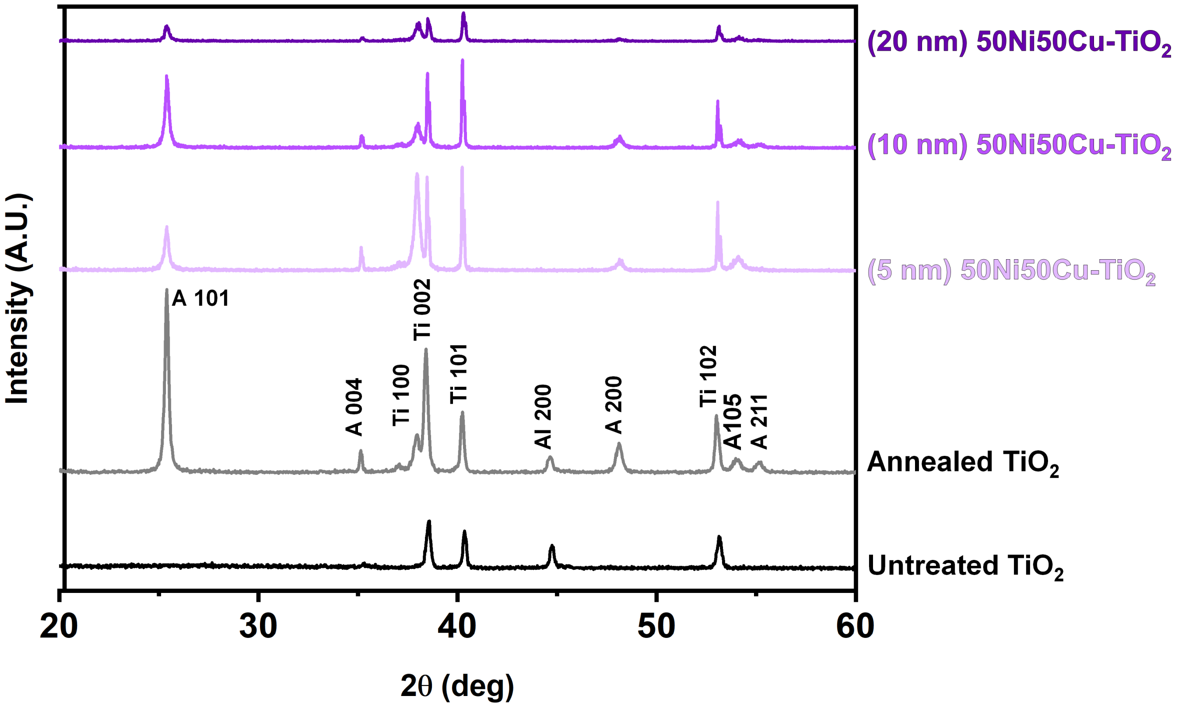

Figure S2. XRD patterns for as-anodized (black line), annealed (grey), and 50Ni50Cu-decorated (purple lines, different nominal thicknesses) photocatalysts.

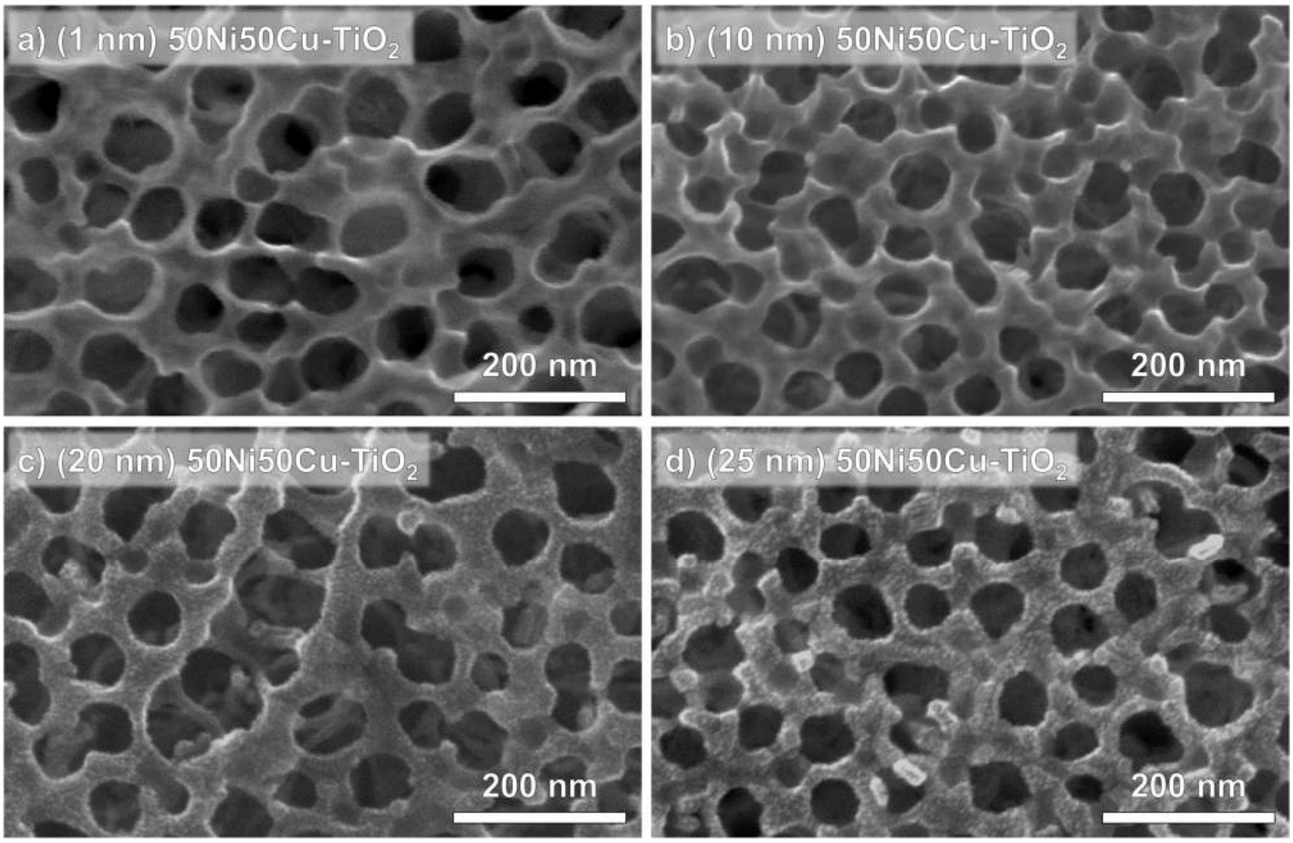

Figure S3. Top-view SEM micrography of a) (1 nm) 50Ni50Cu-TiO₂, b) (5 nm) 50Ni50Cu-TiO₂, c) (10 nm) 50Ni50Cu-TiO₂, and d) (25 nm) 50Ni50Cu-TiO₂.

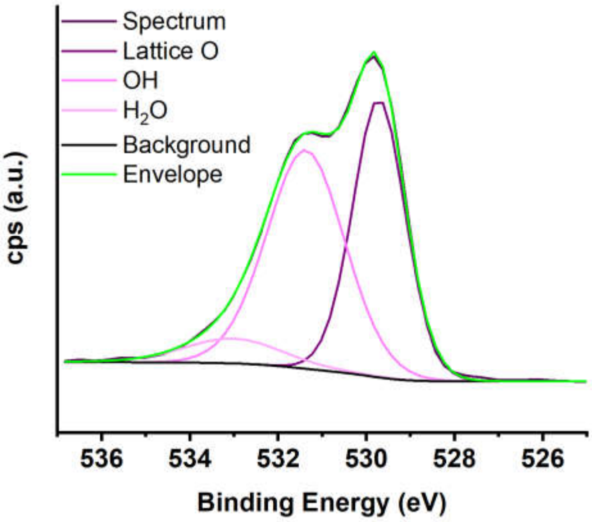

Figure S4. HR-XPS spectrum and fitting in the O1s region for the (20 nm) 50Ni50Cu-TiO₂ sample.

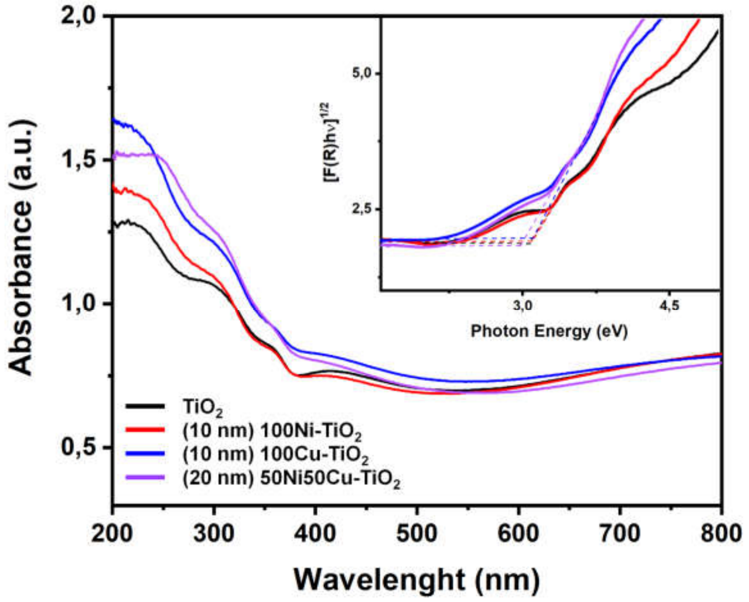

Figure S5. DR-UV-vis absorption spectra of pristine TiO_2 nanotubes (black line), (10nm) 100Ni-TiO_2 (red line), (10 nm) 100Cu-TiO_2, and (20 nm) 50Ni50Cu-TiO_2 (purple line). Inset: Tauc's plots obtained using the Kubelka-Munk method for the different samples. E_g is calculated as the x-axis value of the intercept point between the two linearized segments of the plot.

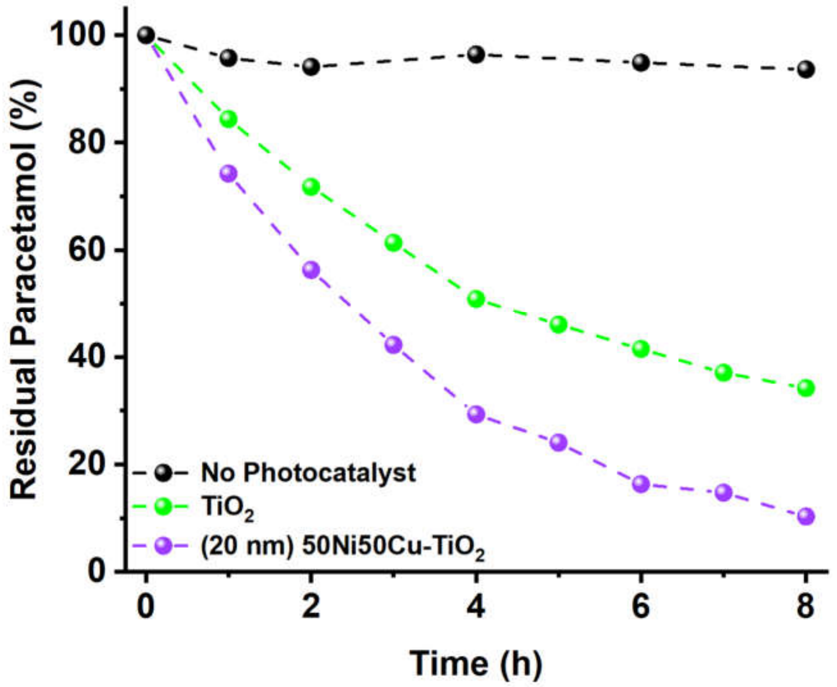

Figure S6. Kinetics test performed under simulated solar radiation by employing TiO_2 (green) and (20 nm) 50Ni50Cu-TiO_2 (purple) as well as a photostability test (black).

Table S3. HR-MS results at different UV irradiation times.

Detected Mass	ESI mode	Brute Formula	Identified Molecule	0 hours	3 hours	8 hours	24 hours
**152.0703**	+	$C_8H_{10}ON$	C_8H_9ON (Paracetamol, a)	Detected	Detected	Not detected	Not detected
**138.0197**	-	$C_6H_4O_3N$	$C_6H_5O_3N$ (4-nitro-phenol, b)	Not detected	Detected	Detected	Detected
**110.0597**	+	C_6H_7ON	C_6H_6ON (4-amino-phenol, c)	Not detected	Detected	Detected	Detected
**109.0298**	-	$C_6H_5O_2$	$C_6H_5O_2$ (Hydroquinone, d)	Not detected	Detected	Detected	Detected
**60.0442**	+	C_2H_6ON	C_2H_5ON (Acetamide, e)	Not detected	Detected	Detected	Detected
